# Binding Strength of Gram-Positive Bacterial Adhesins

**DOI:** 10.3389/fmicb.2020.01457

**Published:** 2020-06-25

**Authors:** Yves F. Dufrêne, Albertus Viljoen

**Affiliations:** Louvain Institute of Biomolecular Science and Technology, Catholic University of Louvain, Louvain-la-Neuve, Belgium

**Keywords:** Gram-positive bacteria, adhesins, physical stress, force, staphylococcus, atomic force microscopy

## Abstract

Bacterial pathogens are equipped with specialized surface-exposed proteins that bind strongly to ligands on host tissues and biomaterials. These adhesins play critical roles during infection, especially during the early step of adhesion where the cells are exposed to physical stress. Recent single-molecule experiments have shown that staphylococci interact with their ligands through a wide diversity of mechanosensitive molecular mechanisms. Adhesin–ligand interactions are activated by tensile force and can be ten times stronger than classical non-covalent biological bonds. Overall these studies demonstrate that Gram-positive adhesins feature unusual stress-dependent molecular interactions, which play essential roles during bacterial colonization and dissemination. With an increasing prevalence of multidrug resistant infections caused by *Staphylococcus aureus* and *Staphylococcus epidermidis*, chemotherapeutic targeting of adhesins offers an innovative alternative to antibiotics.

## Bacterial Adhesion: Force is the Key

Molecular interactions between pathogen adhesins and their ligands play a central role in controlling cell adhesion, the first step leading to infection. Traditional bioassays probe interactions of large ensembles of molecules typically under equilibrium conditions, and provide information on bulk receptor-ligand affinity (dissociation constants). Because most bacterial pathogens are exposed to physical stresses (Berne et al., 2018; Dufrêne and Persat, 2020), it is becoming clear that direct force measurement of adhesin binding strength at non-equilibrium is more relevant than equilibrium methods. During infection, bacteria experience external shear, tension, or compression, meaning understanding how the cells sense and respond to these physical cues has become an important challenge in current mechanobiology (Dufrêne and Persat, 2020). A crucial question is how force controls the adhesive functions of adhesins. The adhesion or unbinding strength is the force at which an adhesin detaches from its ligand with applied load. In most instances, this parameter decreases with applied force (slip bond). However, some adhesion proteins strengthen under tensile load, a behavior called catch bonding (Thomas et al., 2008). The prototypical example of catch bonds is the pilus protein FimH from *Escherichia coli*. This mannose-specific adhesin plays a major role in urinary tract infections by mediating shear-enhanced bacterial adhesion to host cells. Whether catch bonding is a widespread phenomenon among bacterial pathogens remains a controversial issue. Here below we show that there is now compelling evidence that Gram-positive bacteria have evolved force-dependent mechanisms to tune cell adhesion. Strong interaction forces provide the cells with a means to firmly adhere to protein-coated surfaces and to resist high shear stress conditions, while weak forces favor cell detachment and the colonization of new sites. Force is thus a critical parameter of the adhesin function and activity.

**FIGURE 1:**
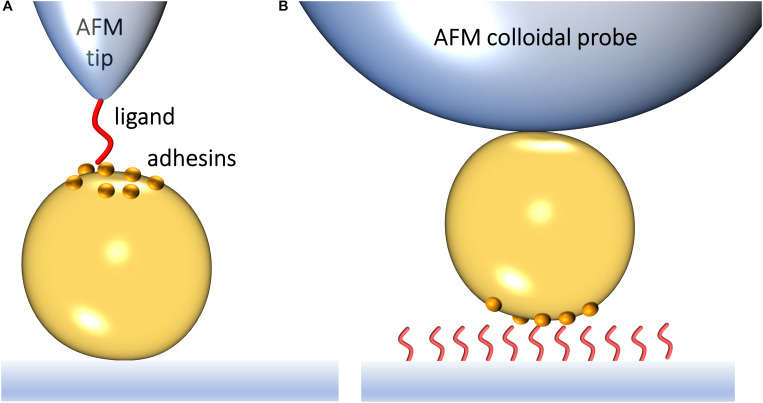
Studying the interaction forces of Gram-positive bacterial adhesins by atomic force microscopy. **(A)** In single-molecule force spectroscopy (SMFS), the AFM probe is functionalized with a specific biomolecule, which can be a purified adhesin or its ligand. Bringing a ligand-functionalized probe into contact with a bacterial surface exposing an adhesin of interest allows capturing the adhesin binding strength upon probe-cell separation. **(B)** In single-cell force spectroscopy (SCFS), a colloidal AFM probe is used to first catch a single bacterium and then probe the strength of interaction between the cell and purified ligands on a substrate or another cell.

Atomic force microscopy (AFM) makes it possible to force probe single adhesins on living bacteria, enabling researchers to identify novel binding mechanisms and to understand how they regulate biofilm formation (Xiao and Dufrêne, 2016). SMFS relies on functionalizing an AFM probe to expose a ligand of interest, such as Fn or Fg (Figure 1A). Bringing the modified probe in contact with and subsequently retracting it from a living bacterium exposing specific adhesins allows to generate an FD curve (Hinterdorfer and Dufrêne, 2006). From the FD plot, the magnitude of the binding strength (or adhesion force) in piconewtons (pN) can be assessed, together with other biophysical parameters. By varying the pulling speed, dynamic force spectroscopy data are generated (Pfreundschuh et al., 2017). Modeling such data with appropriate theories provides quantitative information on thermodynamic and kinetic parameters of the single-molecule interaction. A variation of SMFS is SCFS where a living cell is attached to the probe, thus enabling the measurement of interaction forces between whole cells and protein-coated surfaces or other cells (Figure 1B).

## Staphylococcal Adhesins Under Tension

Staphylococci cause a wide range of infections, including skin and soft tissue infections, bone and joint infections, but also bacteremia and endocarditis. In the latter infections, the bacteria are likely exposed to high fluid shear flow within the vasculature (Guo et al., 1995). The vascular shear flow may allow dissemination of staphylococcal microcolonies, which also have to adhere and resist shear flow (Sherman et al., 2019). The effect of shear on staphylococcal biofilms is thus an important facet of their pathophysiology. Indeed, *Staphylococcus epidermidis* and *Staphylococcus aureus* are notorious for forming biofilms on indwelling medical devices (Otto, 2009). *S. aureus* is equipped with a wide panel of adhesion mechanisms allowing it to evade host immunity (Foster, 2005). Today, highly recalcitrant methicillin-resistant *S. aureus* (MRSA) is regarded as one of the most successful modern pathogens (Turner et al., 2019). Staphylococcal species all express MSCRAMMs, which play crucial roles in adhesion and biofilm formation (Patti et al., 1994; Foster and Höök, 1998; Foster, 2019a,b). Within a few years, AFM has brought fascinating new insights into the molecular mechanisms of MSCRAMMs, showing that force and function are intimately connected in these adhesins.

### Fibronectin-Binding Proteins

Undoubtedly, FnBPs have been the most widely investigated MSCRAMMs so far. Fn is a large extracellular matrix glycoprotein that contains three repeat-domain modules (FnI, FnII, and FnIII) and that is non-covalently anchored to cells through its binding to plasma membrane spanning integrins (Mouw et al., 2014). Gram-positive FnBPs bind *via* C-terminal domains containing tandem repeats to canonical (N-terminal FnI_1__–__5_ modules) and non-canonical (FnI_6_FnII_1__–__2_FnI_7__–__9_ and FnIII_9_,_10_,_12_ modules) sites in Fn (Hymes and Klaenhammer, 2016). Staphylococcal FnBPs, in particular, play multifunctional roles in adhesion by interacting with several ligands including themselves (Foster, 2016). Early investigations reported a weak ∼60-pN force measured for single Fn–FnBP bonds and a linear increase in unbinding force as a function of the loading rate, i.e., the speed at which force is applied (Bustanji et al., 2003). A positive correlation between contact time and adhesion force was observed (Xu et al., 2008), but conflicted on the specific involvement of FnBPs. Later, it was shown that double knock-out of the genes encoding FnBPA and FnBPB in *S. aureus* extinguished binding to Fn-coated AFM probes, while ectopic expression of these two proteins in *Lactococcus lactis*, conferred Fn-binding in this naturally non-Fn-binding bacterium (Buck et al., 2010). Another study revealed a distinct sawtooth-shaped force signature indicative of unfolding of multiple parallel FnI/FnII domains and a zipper array of Fn–FnBP bonds, supported by the absence of such a signature in isogenic mutants (Lower et al., 2010). Mapping the positions of the FnBP–Fn binding signatures showed that FnBPs on *S. aureus* cells were localized at the cell edges close to the Fn-support suggesting that adhesin clustering is induced in response to a primitive prokaryotic tactile surface sensing mechanism (Lower et al., 2010). In the clinical context, *S. aureus* small colony variants isolated from cystic fibrosis sufferers were demonstrated to sustain strong FnBP–Fn interactions via SigB-dependent high-level FnBP expression (Mitchell et al., 2008). In the same line, bloodstream *S. aureus* isolates from patients with cardiovascular implants formed mechanically strong bonds with Fn, involving cluster bonds of up to 80 proteins in parallel (Casillas-Ituarte et al., 2012). Moreover, isolates from patients with infected devices exhibited significantly longer bond lifetimes with Fn than those from patients with sterile devices, which was accounted for by amino acid polymorphisms in Fn-binding domains of FnBPA (Lower et al., 2011). Amino acid changes within high-affinity Fn-binding repeats in FnBPA in *S. aureus* isolated from patients with persistent bacteremia exhibited increased binding strength with Fn and appeared to impart conformational changes in Fn modulating affinity and unbinding (Xiong et al., 2015). Similarly, amino acid polymorphisms within the structured A domain of *S. aureus* FnBPA altered its binding to the abundant blood circulating glycoprotein, Fg (Casillas-Ituarte et al., 2019).

FnBPA is also engaged in homophilic cell–cell interactions, which originate from multiple low-affinity bonds (force of ∼125 pN) between A domains on neighboring cells (Herman-Bausier et al., 2015). The moderate strength of homophilic bonds may be important for biofilm dissemination, by contributing to cell detachment (isolated cells or cell clusters), therefore favoring colonization of new sites. Similar low-affinity homophilic bonds were also observed for an unrelated protein involved in cell–cell interactions and biofilm formation, the Sdr protein, SdrC (Feuillie et al., 2017). On the other hand, Zn^2+^-dependent homophilic interactions between pairs of the *S. aureus* surface protein, SasG, resisted much stronger forces (∼500 pN) (Gruszka et al., 2015; Formosa-Dague et al., 2016). The high mechanostability of SasG is likely to be of biological relevance. Under physical stress, protein unfolding may expose cryptic domains, which together with the rod-like shape of the protein will favor strong intercellular adhesion under flow. Looking at cellular invasion, a recent breakthrough is that the FnBPA–Fn complex binds α5β1 integrins with significantly greater strength than Fn alone, favoring invasion (Prystopiuk et al., 2018). The proposed explanation is that binding of FnBPA to Fn allosterically activates integrin binding to Fn, resulting in strong Fn–integrin interactions.

### Serine-Aspartate Repeat Proteins and Clumping Factors

**FIGURE 2:**
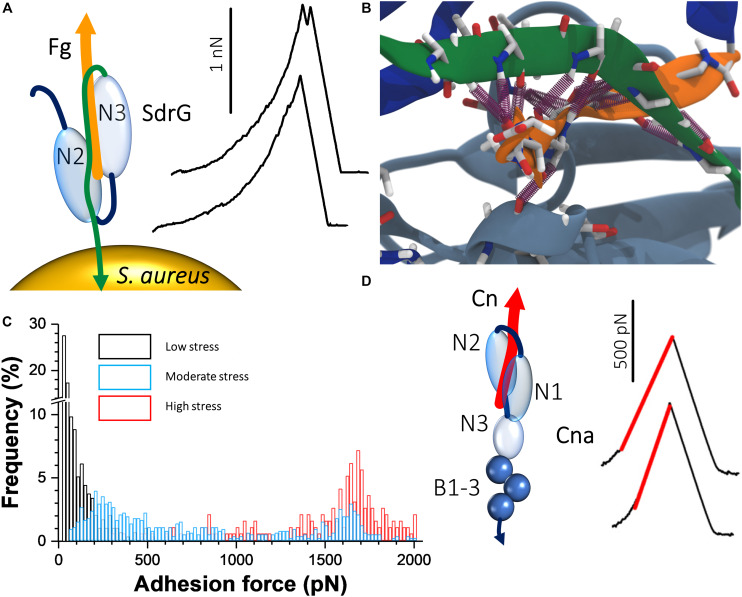
Staphylococcal adhesins can bind their ligands with extremely strong forces. **(A)** The “dock, lock, and latch” mechanism involves docking of Fg on to a sequence between the N2 and N3 domains of SdrG. A C-terminal “locking strand” extension (green) of the N3 domain then folds over the docked Fg, locking it in place and binds to a β sheet within the N2 domain, stabilizing the interaction. On the right are typical force curves for the SdrG–Fg interaction showing that the unbinding force is around 2000 pN. Adapted with permission from Herman et al. (2014). **(B)** The unique mechanical stability of the SdrG–Fg bond is attributed to extensive hydrogen bonding (purple cylinders) formed between the SdrG N2/N3 domains (light and dark blue, respectively) and Fg (orange) and the specific geometry of this molecular interaction under load. A screw-like arrangement of the hydrogen bonds sustains a perfect shear geometry between SdrG and Fg under load. Adapted with permission from Milles et al. (2018). **(C)** Force activation of strong binding between ClfA and Fg. Shown are overlays of histogram plots of adhesion force when the ClfA–Fg bond is subjected to different levels of mechanical stresses by varying the loading rate. Adapted with permission from Herman-Bausier et al. (2018). **(D)** Interaction between collagen (Cn) and single Cna adhesins exposed on living bacteria. The “Cn hug” mechanism involves docking of Cn in a furrow within the Cna N2 domain (left); subsequently, a linker between the N2 and N1 domains folds over the docked Cn and a C-terminal latch sequence of the N2 domain binds within the N1 domain, consequently locking Cn in place. Isopeptide bonds within the C-terminal B repeats of Cna prohibit unfolding under mechanical stress. AFM showed that the Cn–Cna bond behaves like a nanospring when pulled apart, as evidenced by FD curves (right). Adapted with permission from Herman-Bausier et al. (2016).

A remarkable recent discovery is the ultrastrong forces by which staphylococcal Sdr proteins bind to their ligands (Herman et al., 2013, 2014; Milles et al., 2018). The prototypical example is *S. epidermidis* SdrG, which binds via a “DLL” mechanism to Fg (Ponnuraj et al., 2003; Bowden et al., 2008; Foster et al., 2014). A sequence between the N2 and N3 domains within the N-terminal A region of the adhesin “docks” on to a 14-amino acid sequence within the Fg β-chain N-terminus (Figure 2A). Upon stable docking, an extension of the N3 domain folds over the bound Fg peptide, “locking” it in place, and then “latches” on to a β sheet in the N2 domain, stabilizing the SdrG–Fg complex considerably. Single-molecule AFM demonstrated that the SdrG–Fg interaction can sustain forces in the range of 2 nN, the strength of a covalent bond (Figure 2A) (Herman et al., 2014; Milles et al., 2018). Molecular dynamics simulations revealed the underlying molecular mechanism (Figure 2B). The target peptide, confined in a screw-like manner in the binding pocket of SdrG, distributes forces mainly toward the peptide backbone through an intricate hydrogen bond network (Milles and Gaub, 2020).

In addition to the remarkable strength of the SdrG interaction with Fg, SMFS studies also demonstrated that *S. aureus* clinical isolates exhibited an increased density of SdrG on their surfaces, which correlated with increased adhesion on Fg-coated substrates (Vanzieleghem et al., 2015). SdrG thus appears to be a formidable player in staphylococcal adhesion to Fg-coated medical implants. Examples also exist of Sdr proteins that interact with other extracellular matrix proteins, such as *S. epidermidis* SdrF that is responsible for its binding to Cn, which involved weak as well as strong bonds (Herman-Bausier and Dufrêne, 2016).

Another set of staphylococcal MSCRAMM adhesins that are related to the serine aspartate repeat proteins are the Clfs. It was found that the force of ClfA binding to Fg increases sharply from ∼100 pN under low tension to forces exceeding 1.5 nN under high tension (Figure 2C), which was specifically dependent on the C-terminal portion of the Fg γ chain (Herman-Bausier et al., 2018). This shows that ClfA-Fg is activated by mechanical force, reminiscent of a catch bond behavior (Sokurenko et al., 2008). The very strong forces enable the pathogens to resist high shear stress conditions, which often occur during colonization. Analogous to the FnBPA–Fn–α5β1 integrin ternary complex discussed in the previous section, the participation of ClfA in a similar complex involving Fg and the endothelium integrin α_*V*_β3 was investigated and shown to resist strong forces in the 0.8 nN range (Viela et al., 2019). When stress on the ClfA–Fg–α_*V*_β3 complex is minimal, the interaction between Fg and the integrin depends on C-terminal RGD sequences of dimeric Fg α chains. However, under mechanical tension, cryptic N-terminal α chain RGD sequences are exposed that increases the stability of the interaction.

*Staphylococcus aureus* ClfB binds the skin cornified cell envelope proteins cytokeratin and loricrin (Ganesh et al., 2011; Xiang et al., 2012). The force interaction between ClfB and loricrin was investigated and revealed to be mechanically activated, with high forces (1.5 nN range) being sustained under tensile stress (Vitry et al., 2017). Focusing on *S. aureus* adhesion to corneocytes, SCFS studies revealed a dependency on ClfB (Feuillie et al., 2018). Importantly, it was also found that reductions in the levels of natural moisturizing factor in the corneocytes lead to increased adhesion by the bacteria. Interestingly, ClfB binds exclusively to human Fg (Walsh et al., 2008). In the same line, the cell surface protein SpsL from *Staphylococcus pseudintermedius*, which causes disease in dogs, binds with strong forces (up to 2 nN) to canine but not human Fg (Pickering et al., 2019). Overall, these experiments demonstrate the crucial role of protein mechanics in tuning the adhesive functions of bacterial pathogens. Staphylococci have evolved fascinating force-dependent ligand-binding mechanisms that help the cells to attach firmly to biomaterials under high shear stress, and to detach under low shear stress to colonize new sites.

### Collagen Adhesin

The Cn-binding protein Cna plays important roles in bacterium-host adherence and in immune evasion. The strength of Cna–Cn bonds was shown to be very strong (∼1.2 nN; Figure 2D) (Herman-Bausier et al., 2016), consistent with the high-affinity “Cn hug” mechanism, a variation of the high-affinity DLL mechanism. The B region of the adhesin was required for strong ligand binding and functioned as a spring capable of sustaining high forces, potentially due to isopeptide bonds that prohibit unfolding. This mechanical response provides a means to project the A region away from the bacterial surface and to maintain bacterial adhesion under conditions of high forces (Deivanayagam et al., 2000). The force interactions between Cna and C1q, a complement component, and another extracellular matrix protein, laminin, were also investigated (Valotteau et al., 2017). The forces in these interactions were considerably smaller than in the Cna–Cn interactions indicating a different binding mechanism to that of the Cn hug. Interestingly, it was observed that at the single-cell level Cna binding to C1q involved at most two bonds, while in the case of laminin, it involved multiple bonds indicating that multivalency or cooperativity could enhance the strength of Cna-mediated adhesion. These results show that Cna is a multifunctional protein capable of binding to different host ligands through mechanisms that differ from the classical Cn hug.

### Surface Protein A

*Staphylococcus aureus* was also found to adhere to endothelial cells under hematogenous shear flow via interactions with the large multimeric glycoprotein vWF (Claes et al., 2014). The forces in the interaction between *S. aureus* (and MRSA) SpA and vWF were recently unraveled (Viela et al., 2019). Like for several staphylococcal adhesins, the SpA–vWF interaction is force activated and very strong, resisting forces in the 2 nN range. Activation of the SpA–vWF interaction under tension may promote adhesion of bacteria to damaged blood vessels.

## Other Gram-Positive Adhesins

Besides the above studies, there have also been reports on non-staphylococcal Gram-positive adhesins. The SpaA pilus subunit from the nasopharyngeal pathogen *Corynebacterium diphtheriae*, was subject of an SMFS study (Echelman et al., 2016). The results revealed how mechanical energy is efficiently dissipated *via* unfolding and refolding of isopeptide bond-delimited polypeptide loops within the CnaA domains of SpaA. The authors posited that CnaA domains may allow pili to withstand severe forces induced by coughing by dissipating energy away as heat, thus supporting *C. diphtheriae* infection. Focusing on *Streptococcus pyogenes* that causes numerous infections among which pharyngitis and tonsillitis, the forces of the pilus-tip adhesin Spy0125 was investigated (Echelman et al., 2017). It was found that in its folded state, a thioester bond within Spy0125 could be cleaved by nucleophiles, but when the adhesin was put under tension resulting in it being mechanically unfolded, cleavage of the thioester bond could no longer be achieved. In the absence of mechanical stress, cleavage and reformation of the thioester bond was in equilibrium. These results thus indicated that the reversible cleavage of the thioester bond may allow the adhesin to circumvent the activity of inflammation-associated molecules that may attack it, allowing interaction with its host ligands, which under mechanical shear stresses is stabilized.

SpaC, the key pilus adhesion protein of the probiotic Gram-positive bacterium *Lactobacillus rhamnosus* GG (LGG) was shown to feature broad specificity. SpaC formed homophilic trans-interactions engaged in bacterial aggregation and specifically bound mucin and Cn (Tripathi et al., 2013). LGG pili exhibit two unique mechanical responses, that is, zipper-like adhesion involving multiple SpaC molecules distributed along the pilus length and nanospring properties enabling pili to resist high forces. These mechanical properties may be a general trait of Gram-positive pili, enabling the cells to adhere under shear stress conditions.

In the context of tooth decay, where pathogens are exposed to salivary sheer flow (Prakobphol et al., 1995), *Streptococcus mutans* is a major cause of dental caries. *S. mutans* adheres to dental-immobilized SAG. The interaction between the *S. mutans* adhesin P1 and SAG was studied by SMFS and relatively weak forces were observed (∼50 pN) (Sullan et al., 2015). However, SCFS revealed much greater forces in this interaction (∼500 pN) that may indicate binding of multiple P1 molecules to SAG glycoproteins, strengthening *S. mutans* adhesion.

## Targeting Adhesins for Therapy

In a context of increasing drug resistance among Gram-positive pathogens, antiadhesion therapies are attractive because they may supplement waning arsenals of available antibiotics and because they do not target essential processes, they have the added potential advantage of eliciting less and slower resistance acquisition (Krachler and Orth, 2013a, b; Geoghegan et al., 2017; Arciola et al., 2018). Historically, the most widely investigated system is the blocking of the attachment of uropathogenic *E. coli* bacteria to host epithelial cells (Flores-Mireles et al., 2015). Mannosides have been shown to be efficient in inhibiting the adhesion of FimH to host cells (Cusumano et al., 2011). Important lessons were also learned from AFM. Cranberry juice inhibited the fimbriae-mediated adhesion of *E. coli* to solid surfaces and host cells (Pinzón-Arango et al., 2009; Liu et al., 2010; Tao et al., 2011). Similarly, glycofullerenes blocked the force interactions between *E. coli* fimbriae and their carbohydrate receptors (Beaussart et al., 2016).

In the interaction between the *S. aureus* Cna adhesin and Cn, two monoclonal antibodies with competitive inhibitory activity were identified by screening a collection of monoclonal anti-Cna antibodies (Herman-Bausier et al., 2016). Another monoclonal antibody from the same collection blocked the interaction between Cna and the complement protein C1q as well as the extracellular matrix protein laminin (Valotteau et al., 2017). Also, AFM studies revealed the competitive inhibition of *S. aureus* SdrC homophilic interactions by a peptide derived from β-neurexin (Feuillie et al., 2017) and the inhibition by heparin of *S. epidermidis* clinical isolates’ adhesion to Fn (Bustanji et al., 2003). Notably, the resolution of the crystal structure of ClfA in complex with the monoclonal antibody tefibazumab offers the potential for the rational design of antiadhesive antibodies targeting staphylococci (Ganesh et al., 2016), in which AFM could serve as a platform to study structure-activity relationships. Accordingly, AFM may be used to screen novel compounds with antiadhesive properties as well as to decipher their mechanisms of action.

Lastly, AFM is also a valuable tool to unravel how antimicrobials alter the specific and non-specific adhesion forces of bacteria. AFM helped unravel the interplay between staphylococcal adhesion to solid surfaces and cell wall deformation under treatment with cell wall active and non-active antibiotics (Carniello et al., 2018). In mycobacteria, the efficient first line antitubercular, ethambutol, had a strong effect on *Mycobacterium bovis* BCG–Fn interactions (Verbelen and Dufrêne, 2009), while several compounds targeting mycolic acid biosynthesis strongly decreased the magnitudes and frequencies of hydrophobic adhesive forces measured on *M. bovis* BCG or *Mycobacterium abscessus* cells (Alsteens et al., 2008; Viljoen et al., 2020). AFM also disclosed the inhibitory effect of the herbicide 2,4-dichlorophenoxyacetic acid on *E. coli* non-specific adhesion (Bhat et al., 2015, 2018a,b). Interestingly, SMFS studies detected the massive surface exposure of *Candida albicans* Als adhesins after treatment with the antifungal caspofungin (El-Kirat-Chatel et al., 2013). Homophilic interactions between Als adhesins play an important role in cellular aggregation (Dehullu et al., 2019), highlighting the importance of studying the effects of antimicrobials on adhesion.

## Outlook

The discoveries discussed here represent an important step forward in our understanding of the molecular mechanisms used by Gram-positive pathogens to mediate cell adhesion and trigger infections. AFM experiments have shown that Gram-positive bacterial adhesins feature a wide range of binding strengths, from ∼50 to more than ∼2000 pN. A remarkable finding of the past years is that some adhesins bind their ligands with extremely strong forces that are activated by mechanical tension, as in catch bonds, an intriguing phenomenon that enables the pathogens to firmly bind to host cells and protein-coated surfaces, and to sustain high shear stress. For years, the only well-investigated catch bond behavior was the mannose-binding pilus-tip adhesin FimH from the Gram-negative *E coli*. We are now starting to realize that Gram-positive staphylococcal adhesins might become a new paradigm in catch bond adhesion. These binding mechanisms represent potential targets to fight infections, and AFM might become a valuable tool for the screening of antiadhesion compounds like small peptides and antibodies.

## Author Contributions

YD and AV contributed equally to the writing of this manuscript.

## Conflict of Interest

YD is a Research Director at the FNRS. The remaining author declares that the research was conducted in the absence of any commercial or financial relationships that could be construed as a potential conflict of interest.

## Abbreviations

Clf: clumping factor
Cn: collagen
Cna: Cn adhesin
DLL: dock lock and latch
FD: force–distance
Fg: fibrinogen
Fn: fibronectin
FnBP: Fn-binding protein
MSCRAMM: microbial surface components recognizing adhesive matrix molecules
SAG: salivary agglutinin
SCFS: single-cell force spectroscopy
Sdr: serine-aspartate repeat
SMFS: single-molecule force spectroscopy
SpA: surface protein A
vWF: von Willebrand factor.
